# Detection and diagnosis of diabetic eye diseases using two phase transfer learning approach

**DOI:** 10.7717/peerj-cs.2135

**Published:** 2024-09-19

**Authors:** Vamsi Krishna Madduri, Battula Srinivasa Rao

**Affiliations:** School of Computer Science and Engineering (SCOPE), Vellore Institute of Technology-AP University, Amaravati, Andhra Pradesh, India

**Keywords:** Early diagnosis, Defected region, Diabetic eye disease, Retinal fundus images, Deep learning, Transfer learning

## Abstract

**Background:**

Early diagnosis and treatment of diabetic eye disease (DED) improve prognosis and lessen the possibility of permanent vision loss. Screening of retinal fundus images is a significant process widely employed for diagnosing patients with DED or other eye problems. However, considerable time and effort are required to detect these images manually.

**Methods:**

Deep learning approaches in machine learning have attained superior performance for the binary classification of healthy and pathological retinal fundus images. In contrast, multi-class retinal eye disease classification is still a difficult task. Therefore, a two-phase transfer learning approach is developed in this research for automated classification and segmentation of multi-class DED pathologies.

**Results:**

In the first step, a Modified ResNet-50 model pre-trained on the ImageNet dataset was transferred and learned to classify normal diabetic macular edema (DME), diabetic retinopathy, glaucoma, and cataracts. In the second step, the defective region of multiple eye diseases is segmented using the transfer learning-based DenseUNet model. From the publicly accessible dataset, the suggested model is assessed using several retinal fundus images. Our proposed model for multi-class classification achieves a maximum specificity of 99.73%, a sensitivity of 99.54%, and an accuracy of 99.67%.

## Introduction

Patients with diabetes are among the most common disease groups in the world today (Arslan & Erdaş, 2023). Visual loss can result from diabetic fundus disorders, the primary cause of blindness. Visual function is currently affected by fundus illnesses such as glaucoma, cataracts, and diabetic retinopathy (DR) (Raiaan et al., 2023). A patient’s eyesight is severely impaired, and no specific treatment is available when fundus disease reaches a late stage (Sengar et al., 2023). It is challenging to use retinal fundus images to effectively diagnose diabetic retinopathy since even highly competent eye experts frequently misidentify eye abnormalities. It is advantageous to support a method that helps detect diseases since early detection of diseases prevents blindness (Channa et al., 2023). Fundus imaging provides a less expensive and non-invasive alternative to expensive optical coherence tomography (OCT) imaging technologies for identifying and diagnosing ocular disorders (Aranha, Fernandes & Morales, 2023).

For ocular diseases, recent advancements in retinal imaging have prompted researchers to concentrate on computer-aided diagnostic (CAD) systems, such as the integration of CNN with various metaheuristic optimization algorithms (Modi & Kumar, 2023). These CAD systems aim to assist specialists by improving the process of disease diagnosis (Patil et al., 2024). A fundus picture is a projected, two-dimensional, RGB image that mimics the three-dimensional structure of the human retina. It shows the key elements needed to identify ophthalmological illnesses early (Joshi, Sharma & Dutta, 2024). Early retinal disease identification prevents patient blindness (Albelaihi & Ibrahim, 2024; Mondal, Mian & Das, 2021). Anomalies that develop on or around the unique biological components of the human retina are utilized to monitor how ophthalmological diseases progress (Yao et al., 2021). Various anatomical features and issues, including drusen, exudates, and haemorrhages (Vadduri & Kuppusamy, 2023; Ausayakhun et al., 2021), are identified using fundus images with their three coloured channels (red, green, and blue).

Most ophthalmological image analysis research is focused on a small number of illnesses, such as cataracts, glaucoma, dry eye disease, and diabetic macular edema (DME) (Sangeetha et al., 2023) only. This research aims to create a classification model for automated fundus images that can identify a greater variety of ophthalmological illnesses (Alhajim, Al-Shammar & Kareem Oleiwi, 2024; Feng et al., 2023). Fundus images are used in the proposed model for multi-class classifications of diabetic eye diseases (DEDs). In real-life scenarios, anomalies associated with one or more disorders may appear in one or both eyes (Kumar & Singh, 2023). While the left eye appears normal, the right eye may have abnormalities related to diabetic retinopathy (Liu et al., 2023). The suggested model incorporates information obtained from patients’ fundus images. The proposed method takes advantage of effective and broadly applicable deep-learning techniques for classification (Kaur & Kamra, 2023). In the context of ophthalmological diseases, two-phase transfer learning based on convolution neural network (CNN) models that have already been trained is proposed to classify the numerous classifications of fundus images and segment the defective regions (Gour & Khanna, 2021; Wang et al., 2022).

By unlocking and fine-tuning individual layers, the model can focus on learning disease-specific features while retaining the overall knowledge gained during pre-training; Research employing two-phase transfer learning has demonstrated cutting-edge outcomes in tasks about the identification and categorization of diabetic diseases. Compared to models trained from scratch or with single-phase transfer learning, this method has been shown to have better performance; By placing research in this research, it becomes evident that the two-phase transfer learning approach presents a viable means of enhancing the precision and efficacy of diagnosing diabetic eye disease, ultimately leading to improved patient outcomes and care in the fields of ophthalmology and diabetes management.

The remaining sections of the research are described as follows; the literature studies of the existing approaches to diabetic eye diseases are provided in “Literature Survey”. Models for classifying and segmenting fundus images based on two-phase transfer learning are presented in “Proposed Methodology”. “Result and Discussions” confers the evaluation parameters and outcomes. The concluding research is provided in “Conclusion”.

### Novelty of this research

The most significant contributions of this research are,

- This research proposes a two-phase transfer learning approach for classifying and segmenting multi-class DED diseases.
- By removing undesired abnormalities, image pre-processing techniques improved the quality of the images. The unwanted noise could be removed from the grayscale image using median filtering. The image contrast was enhanced by utilizing the Contrast-Limited Adaptive Histogram Equalization (CLAHE) method.
- Effective classification is achieved using the transfer learning-based Modified ResNet-50 model, which classifies the fundus efficiently, including diabetic retinopathy, glaucoma, DME, and cataracts.
- After classification, it is challenging to identify diabetes-related eye disorders using machine learning-based segmentation. The transfer learning-based DenseUNet model is used in the proposed system for the localisation of disease regions. The segmentation results provide precise identification of the affected areas that guarantee accurate disease identification effectively.
- The proposed approach is assessed using publicly accessible datasets. The Python platform is utilized to evaluate the proposed approach. According to the experimental results, out of all the approaches, the proposed approach outperforms the state efficiency.

## Literature survey

DED disease diagnosis has been the subject of several kinds of research conducted to assist medical professionals in determining an early diagnosis. However, in recent times, multiple artificial intelligence techniques have been used to detect and diagnose different classifications of DED diseases.

Sarki et al. (2021a) proposed the transfer learning-based CNN for ocular disease classification. Two alternative methods, such as Model 1 and Model 2, were used while classifying images. Model 1 used two inputs, whereas Model 2 used concatenated inputs while employing transfer learning. With the use of Adam and SGD optimizers, Model-1 was updated. Two different optimizers and four different pre-trained CNN models were used. With SGD optimizer, the performance of the VGG16 pre-trained architecture was improved for fundus image classification.

The novel deep learning-based CNN model was introduced by Demir & Taşcı (2021). The proposed network model is integrated with several image-processing approaches. For image enhancement and segmentation, several image processing methods are combined in this article, and after that, the images are trained in deep learning algorithms.

For multiple classifications of ophthalmologic diseases using fundus images, an effective region-based convolutional neural network and long short-term memory (R-CNN+LSTM) based model was developed by Indumathi, Kalanjiyam & Ramalakshmi (2021). This research successfully classified eight different ophthalmologic disorders. Fundus images were processed using the suggested R-CNN+LSTM structure to extract deep features. Using the NCAR feature selection approach, the relevant features were selected. For the classification phase, the support vector machine (SVM) classifier was used.

For the early detection of diabetic retinopathy, a deep learning-based approach was proposed by Nazir et al. (2021). In their experiment, the fundus images were used to discern diabetic retinopathy. By using the EfficientNet model, the severity of diabetic retinopathy was assessed. The fundus images are grouped into five classifications according to the disease’s severity. For diabetic retinopathy identification, a deep learning method was developed and validated using fundus images.

Joans (2021) proposed a praxis deep learning-based CenterNet model for retinal disease stratification. At first, a specific region of interest was located for suspected samples to generate annotations. At the same time, the CenterNet model was trained over annotated images as part of the proposed approach. The one-stage detector on DenseNet-100 was used specifically for feature extraction, and then the disease lesions were located and categorized using CenterNet.

Fayyaz et al. (2023) proposed the novel CNN model for multiple eye disease classification. Diabetic lesions, glaucoma, and macular oedema are classified in this article. The DED diseases were effectively identified and classified by passing the input images to the various layers of the CNN model. Convolutional layers are used for feature extraction. For fundus image detection based on severity, AlexNet and Resnet101-based feature extraction is utilized by Novitasari et al. (2022) to construct a deep-learning network. Ant Colony systems and interconnected layers both assist in choosing features and identifying the most important features or attributes. The final classification model with a promising level of accuracy was produced by passing these selected features through SVM with several kernels.

To identify DR severity, a hybrid CNN-DELM approach (CDELM) was created by Sarki et al. (2021b). DenseNet, ResNet-101, ResNet-50, ResNet-18, and GoogleNet were the CNN architectures utilized for feature extraction. The DELM method was used to classify the learning result features further. The above-stated opposite works are catalogued in Table 1. Moreover, securing the health care system and storing disease risk assessments with high precision can be formidable if the image classification strategies were integrated with Internet of Things (IoT) based medical systems (Xu et al., 2021; Sharma et al., 2022).

**Table 1:** Literature survey.

Reference	Dataset	Model	Benefits	Difficulties
Gour & Khanna (2021)	ODIR	Transfer learning based VGG-16	(i) It solves the overfitting problem of the network (ii) It reduces the trainable parameters significantly	(i) Class imbalance occurs during the evaluation (ii) The classification performance is decreased
Sarki et al. (2021a)	DRISHTI-GS, Messidor-2, messidor and retinal dataset	CNN	(i) The diagnosis of this model is made accurately and quickly (ii) It reduces the computation costs	(i) It has poor reliability and interpretability of the network model (ii) It was unable to provide valuable and efficient outcomes for diagnosing the disorders
Demir & Taşcı (2021)	ODIR	R-CNN+LSTM	(i) It effectively selects multi-level features (ii) It increases the classification performance	(i) Fast prediction results require powerful hardware (ii) Complex training
Indumathi, Kalanjiyam & Ramalakshmi (2021)	Kaggle	EfficientNet	(i) It accurately classifies images (ii) It generates reliable solutions with high sensitivity and specificity	(i) It reduces the quality of classification in real-time applications (ii) It requires additional time for training
Nazir et al. (2021)	APTOS-2019 and IDRiD	CenterNet	(i) It can easily recognize small lesions and deal with over-fitted training data (ii) It is proficient in correctly locating and classifying disease lesions	It will decrease its classification accuracy in cases where the number of classes are very high
Joans (2021)	Messidor	CNN	(i) It detects the images more fastly (ii) It effectively captures interscale variations	(i) It does not accurately classify low-intensity and noisy data images (ii) Require high computing resources for training on high-resolution data
Fayyaz et al. (2023)	Kaggle	SVM	It helps to identify the significant features of the images	The network model is unreliable and difficult to understand.
Novitasari et al. (2022)	Messidor	CDELM	It is capable of handling over-fitted training data and small lesions with efficiency.	It was unable to provide valuable and efficient outcomes for diagnosing the disorders.

### Research gaps of existing models

- Most existing deep learning-based models fail to detect and characterize small lesions or early-stage pathologies in fundus images, as early detection is crucial for effective treatment.
- For multi-class classification of diabetic eye diseases, several prior models require high computing resources and are not applicable for accurate word settings.
- Most deep learning models for fundus image analysis are considered black boxes. Research is needed to make these models more interpretable, especially for clinical adoption.
- The performance results of the segmentation for accurate lesion segmentation are decreased in most of the existing deep-learning models.

To solve the research gaps in the existing deep learning models, an efficient combined deep learning-based classification and segmentation model is proposed in this research. In the proposed approach, a reliable and effective two-phase transfer learning model is developed in this research.

### Solutions of proposed research

The research employs a two-phase transfer learning approach to address the issues of class imbalance and data over-fitting. By training on high-resolution data with fewer resources and utilizing a smaller batch size, the issue of high computational resource requirements for training on high-resolution data is resolved in this research. Image pre-processing using an effective filter and contrast enhancement technique was employed to categorize low-intensity and noisy data images; it generates precise segmentation and classification outcomes. Effective pre-processing filters and hyper-parameter tuning are used in this research to overcome the issue of an unstable network model, which can significantly enhance the model’s performance and dependability.

In the segmentation network model, we used a dropout layer. The dropout layer randomly sets a fraction of the neurons to zero during each training iteration, which helps prevent the co-adaptation of neurons and reduces the over-fitting problem. We used a minimum number of layers in the classification and segmentation models; the network over-fitting is avoided based on this smaller number of layers. We set the batch size of 20 for training; smaller batch sizes introduce more noise into the training process, which can help prevent over-fitting. We used effective hyper-parameter optimization for network training to avoid over-fitting the network. We used transfer learning with pre-trained models for classification and segmentation; it is used to enhance the generalization abilities of the models and solve the over-fitting problem.

## Proposed methodology

Instead of simple matrix multiplication, a convolution operation is used by the basic CNN architecture. CNN architectures provide effective performance in medical image processing fields such as image classification, image enhancement, and image segmentation. A CNN structure is composed of various processes and layers. Fully connected layers, pooling layers, batch normalization (BN), Rectified Linear Unit (ReLU), and convolutional layer (Conv) are the essential layers in the CNN architecture. To address complex problems, these models require an extensive database. A comprehensive database like ImageNet trains the CNN models to achieve cutting-edge classification performance. For image classification of the ImageNet database, various CNN designs, namely R-CNN, CenterNet, CNN, VGG16-SGD, FPOA-CNN, and Inception v3, are suggested in previous works. Gathering a sizable amount of labelled data is complex and time-consuming for experts. Transfer learning leverages data from previously trained models to form new models, regardless of the domain for which the models were initially generated.

The proposed research used transfer learning using the latest CNN architectures for fundus image classification. Architectures of various sizes were selected and analyzed to more effectively analyze the domain-specific fundus images combined with nature images from the ImageNet collection. In this research, we develop a two-phase transfer learning approach for classifying and segmenting multi-class macular diseases using fundus images.

To align the input dimension of the proposed model, the dataset’s images were initially pre-processed. This was accomplished by setting the input images’ resolution to 224 × 224. Furthermore, the normalization process was applied to the fundus images to prevent the model from overfitting. Pre-processing was initially done to remove undesired distortions and improve image quality. At first, the grayscale image’s unwanted noise was eliminated using median filtering. Then we applied the CLAHE technique to the image enhancement. For retinal disease with multiple classes of classification phase, the pre-processed images are sent into the transfer learning-based pre-trained Modified ResNet-50.

**Figure 1:**
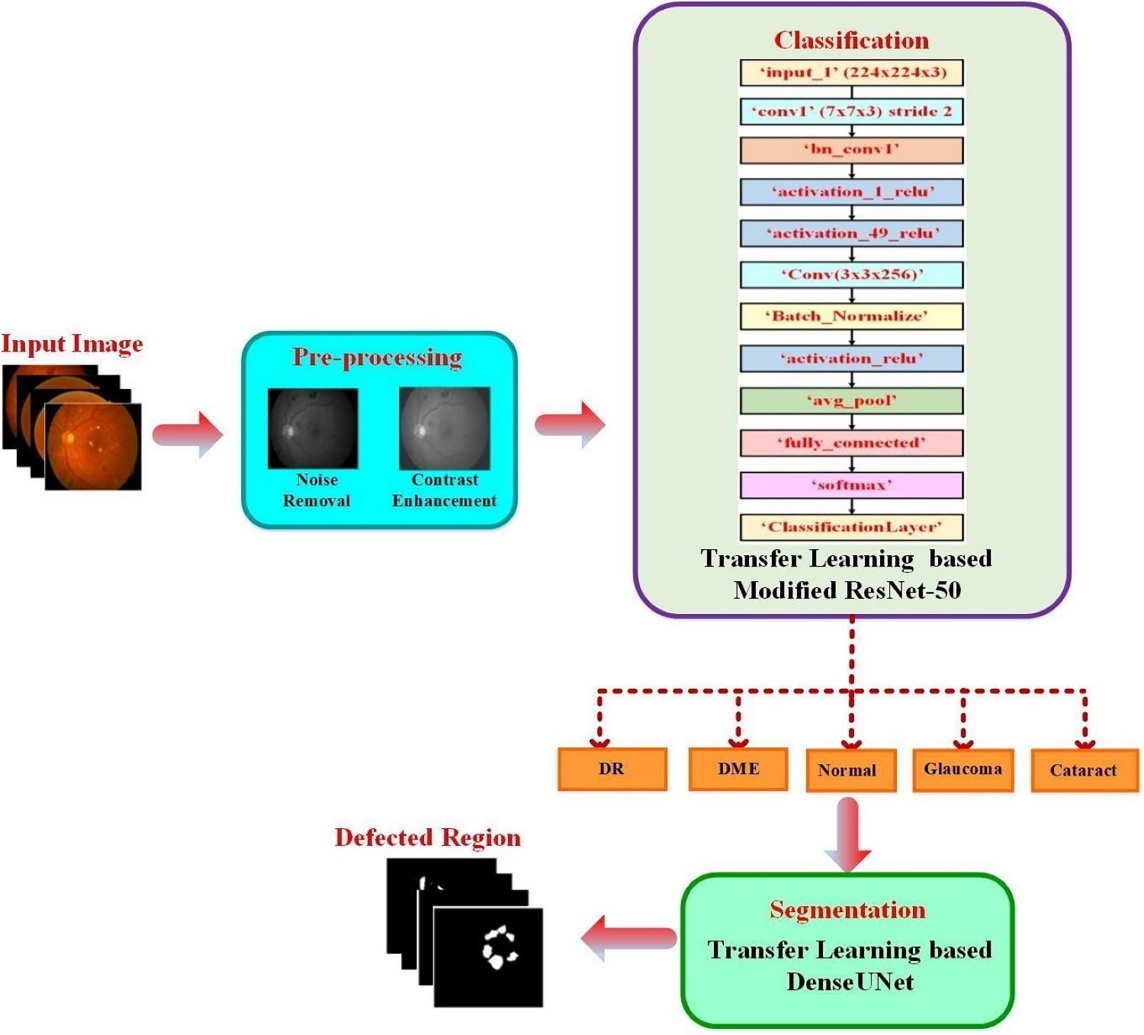
Structure of the proposed two-phase transfer learning approach.

The proposed approach classified the fundus images into typical diabetic retinopathy, DME, cataract, and glaucoma. A transfer learning-based pre-trained DenseUNet model was employed after classification to segment the defected area. The structure of the proposed two-phase transfer learning approach is presented in Fig. 1.

### Dataset collection

**Figure 2:**
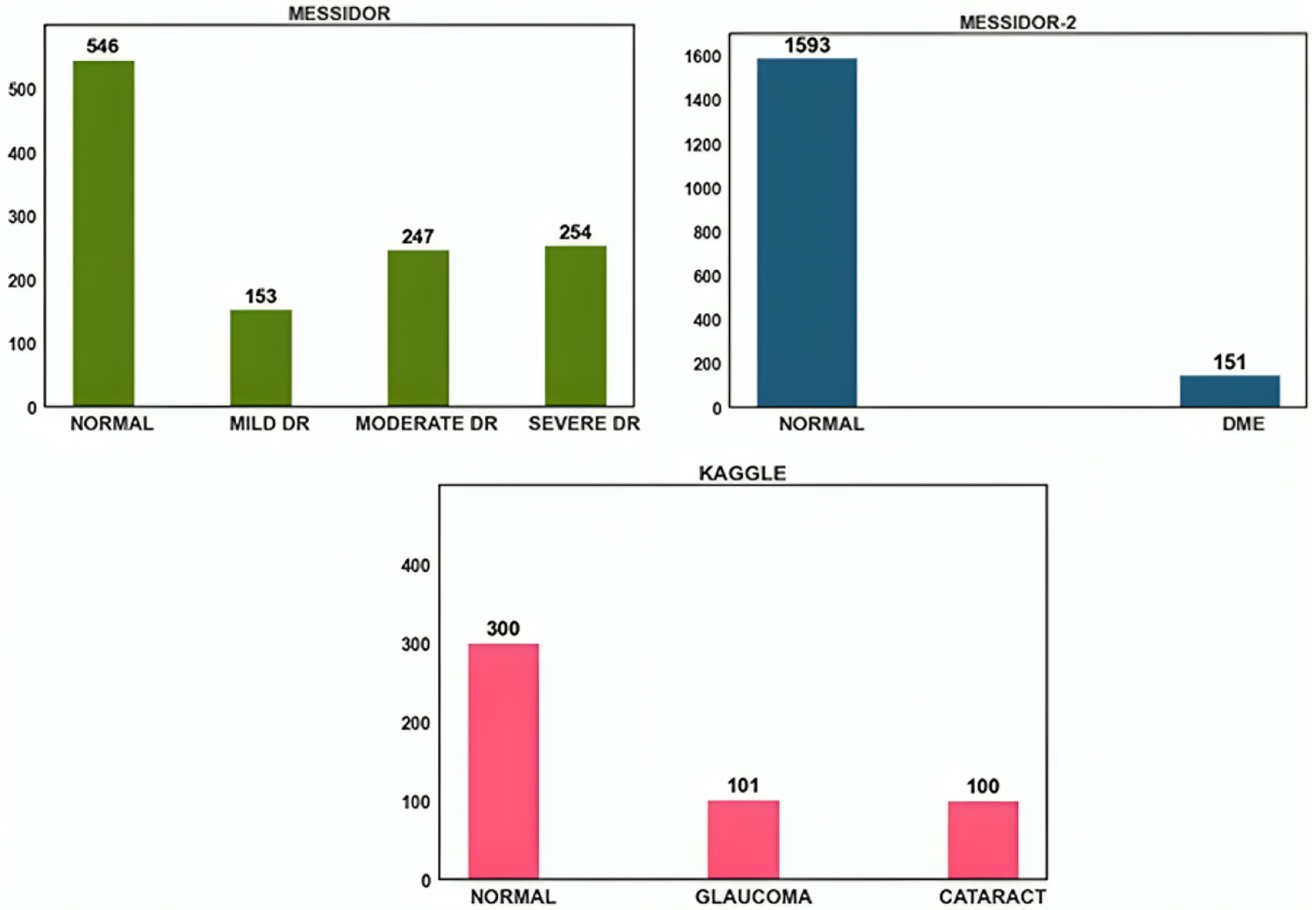
Data distribution of Messidor, Messidor-2, and Kaggle datasets.

The open-source datasets were gathered from DRISHTI-GS, Messidor-2, Messidor, and Kaggle cataract datasets. Despite their modest size, the images in the Messidor dataset were of excellent resolution and had precise categorization. A similar public dataset, Messidor-2, was used by others to analyze the output of the DED algorithm. A total of 1,748 images representing 874 subjects make up the data. Messidor-2 differs from Messidor’s original 1,200-image data set for each eye. Glaucoma and cataract datasets were acquired from the retina dataset, and Kaggle was used to obtain this dataset. Glaucoma and cataracts were each represented by 100 images in this dataset. The dataset distribution is shown in Fig. 2.

### Data pre-processing

#### Noise removal

This study used various image processing approaches to improve the image features. The blurry and low-contrast fundus images were obtained due to speckle noise. Eye movements were the primary source of speckle noise during image capture. In the fundus images, the speckle noise is reduced using median filtering. A median filter produced impressive results for noise reduction and operated at the maximum speed. The medium filtering considers each neighbouring pixel's value to determine if a pixel shows consistency with its surrounding pixels. Also, the noise pixel’s value was updated by using a surrounding pixel value's medium.

#### Image contrast enhancement

The information value and appearance of the original images were enhanced by using image-enhancing techniques before processing. Image visibility was improved by using CLAHE. The Adaptive Histogram Equalization (AHE) technique included a modified CLAHE component. This approach introduced the boosting function to all nearby pixels and derived the transformation function. Due to the contrast amplification limit, CLAHE exhibits less contrast than AHE. In CLAHE, discrete data regions called tiles were used to improve the image’s contrast instead of applying CLAHE to the entire image. Bilinear interpolation was then used to fuse the generated adjacent tiles back together correctly. Grayscale retinal photos were processed with CLAHE. Image noise was reduced by using the “clip-limit” function. The grey level mapping was created and clipped to the histogram. Each grey level in the pixel numbers of the contextual area was divided equally, resulting in the following average number of gray pixels:

(1) $${n_{avg}} = \displaystyle{{{n_{CR - {x_p}}}*{n_{CR - {y_p}}}} \over {{n_{gray}}}}$$

where the average number of pixels is denoted as ${n_{avg}}$.

The number of gray levels in contextual region is represented as ${n_{gray}}$.

Number of pixels in the x direction of contextual region = ${n_{CR - {x_p}}}$.

Number of pixels in y direction of contextual region = ${n_{CR - {y_p}}}$.

Equation (1) provides a primary indication of the image’s resolution. Higher pixel counts generally result in higher-resolution images, which can be crucial in the image classification and segmentation process.

After that, the clip limit is calculated as follows:

(2) $${n_{CL}} = {n_{CLIP}}*{n_{avg}}.$$

The purpose of the clip limit Eq. (2) in image processing is to provide a mechanism for adaptive contrast enhancement. It allows CLAHE to enhance the local contrast of different regions in an image while avoiding over-enhancement or amplification of noise.

Because CLAHE is so good at making the typically conspicuous areas that are generally difficult to access more accessible, it is a valuable tool in biomedical image processing.

### Classification using modified ResNet-50

The classification of the DED dataset was incorporated by employing pre-trained CNNs in this research. The specific feature representations were obtained by converting a feature vector with a specified weight matrix in deep CNN without any gaps in the spatial arrangement’s information. The idea applies features discovered while working on the original task to the targeted jobs. Research areas using significant data and computing resources benefit from transfer learning. The details of the pre-trained models are presented in this section.

We propose a new pre-trained CNN architecture based on a modification to the ResNet50 architecture. In the ResNet50 model architecture, additional layers are added to better fit the fundus image collection. Low-resolution fundus images are taken, and the height-to-width ratio may vary. To implement a similar approach in the developed model architecture, the images in the dataset are resized into 224 × 224 × 3 for training and testing.

**Figure 3:**
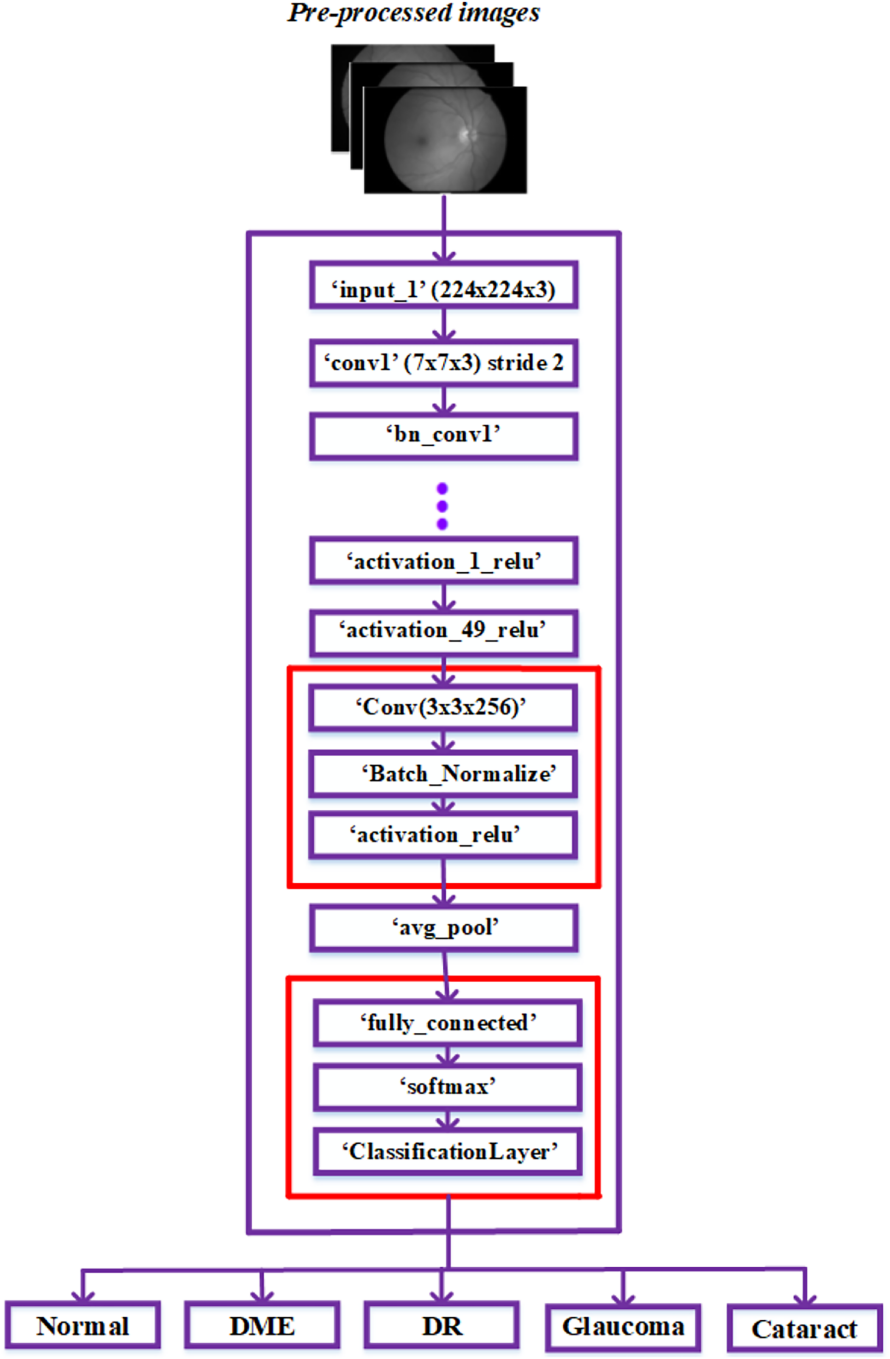
Modified ResNet-50 architecture.

As ResNet could achieve greater accuracy and was relatively simple to improve, it has an excellent deep-learning architecture. Furthermore, the network’s skip connections resolve persistent vanishing gradient problems. The network’s time complexity increases proportionately to the deep network architecture’s layer count. To lessen this complexity, consider using a bottleneck design. As a result, we chose to construct our framework using the ResNet 50 model and ignored other networks with more layers. The architecture is explored in more detail below in Fig. 3.

The ResNet50 architecture has been modified to classify retinal diseases and achieve adequate performance. The last three layers of the ResNet50 architecture, including the classification layer, softmax layer, and fully connected layer, were first modified for our classification process. Our modified network used The fully connected layer instead of the convolution layer. The output size is represented based on the five classes: regular, diabetic retinopathy, glaucoma, DME, and cataracts.

The three layers, convolution, batch normalization, and Activation Relu, were added next in the basic ResNet-50 model. These three layers were used in fundus images to extract robust features automatically. These three layers are added using the procedures listed below.

- The ‘activation_49_relu’ layer is connected to the newly added ‘Conv’ layer and disconnected from the ‘avg_pool’ layer.
- The recently added ‘activation_relu’ layer is connected to the ‘avg_pool’ layer.
- The classification, softmax, and fully connected layers are the last three recently added layers; these layers are mentioned after the “avg_pool” layer in the sequence of appearance.

After injecting the new layers, the ResNet-50 network model’s modified architecture appears in Fig. 3. In the dataset, the image features were obtained by passing the pre-processed images to layers of this modified network. Then, the classification layer of this network classified the fundus images into typical diabetic retinopathy, glaucoma, DME, and cataract. The proposed model was developed to classify a variety of retinal disorders.

### Segmentation

**Figure 4:**
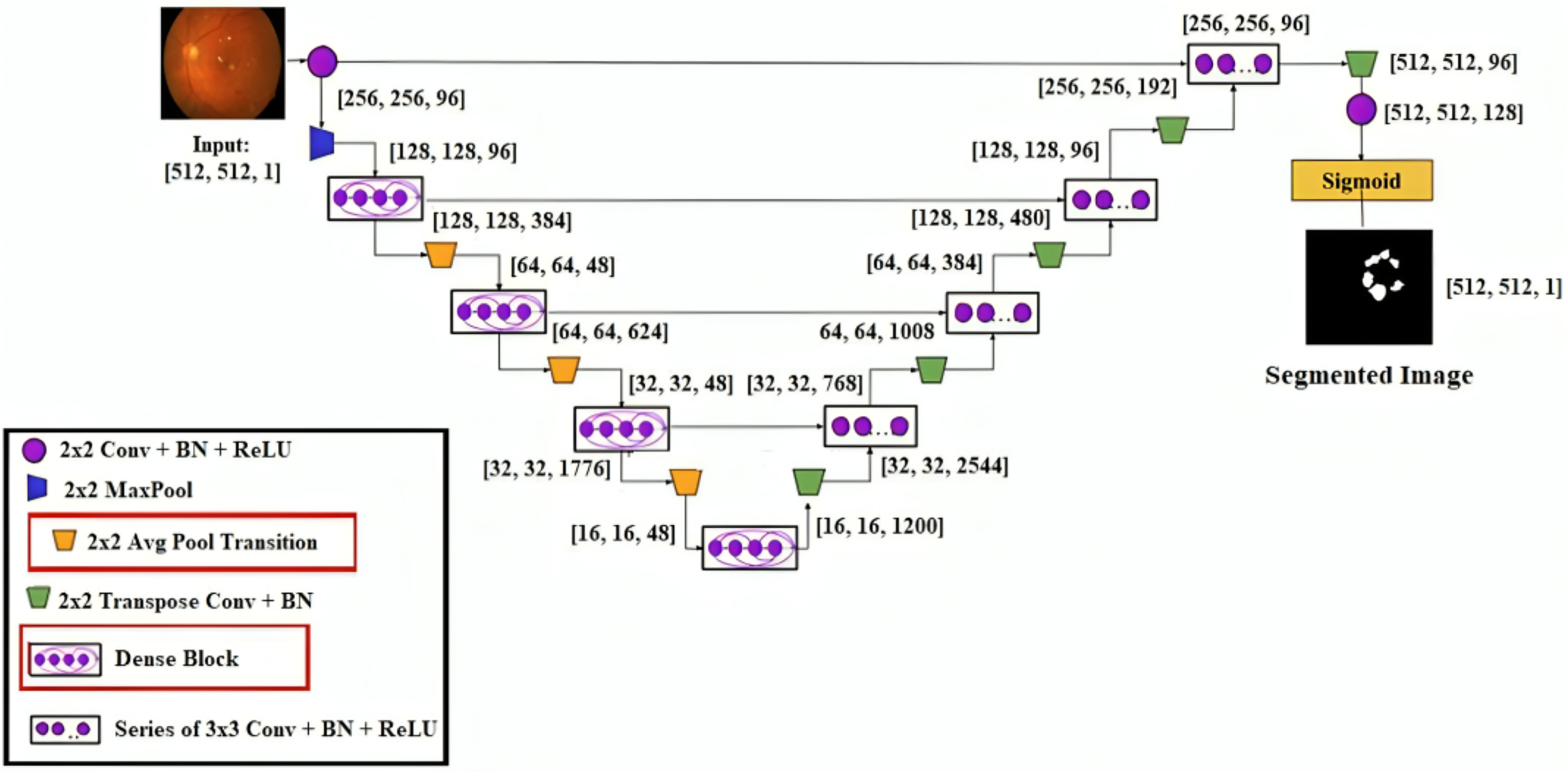
DenseUNet segmentation model’s architectural design.

This research developed a transfer learning-based DenseUNet network model to more precisely segment DED diseases. To segment semantically, we add U-Net to the denseness model. Figure 4 shows the design of the DenseUNet model. The segmentation network model combined the FCN and u-net model’s encoder-decoder design concepts (Kaur & Kaur, 2022). This included an up-sampling path and a down-sampling path. The Input image’s semantic information was extracted by downsampling path using down-sampling and continuous convolution operations layer by layer.

Up-sampling and de-convolution were used to increase the feature map’s resolution until the original resolution of the input pictures was reached. The transition layer and dense block comprised the down-sampling and up-sampling paths. These paths connected adjacent dense blocks. The thick blocks and four transition layers are included in both down-sampling and up-sampling paths. The exact resolution for n consecutive convolution layers was present in the dense block, followed by BN, ReLU, and dropout layers.

Each layer’s input yielded the output feature maps from all previous layers. It is known as dense concatenation. With the same feature size, the layer’s feature maps must contain dense blocks for this process. The proposed DenseUNet architecture consisted of 10 dense blocks. In both the dense down-sampling up-sampling paths, five dense blocks are used. Four densely connected layers were present in each dense block with the same feature size.

They are introducing a transition block for the layer transition. The five layers are used to form the dense down-sampling path. The thick and transition blocks are presented in the dense down-sampling path. The up-sampling layer, merger operation, and thick block operation reconstruct the high-resolution images in the dense up-sampling path. Five layers made up the path, and these layers localised the regions, which also recovered the entire input resolution.

The output of the ${l^{th}}$ layer is defined by ${x_l}$. From the previous layer output ${x_{l - 1}}$, transformation ${H_l}\left( x \right)$ computes the ${x_l}$,

(3) $${x_l} = {H_l}\left( {{x_{l - 1}}} \right).$$

A sequence of operations is represented by ${H_l}\left( x \right)$ convolution (Conv), pooling, BN or ReLU, *etc*. To avoid vanishing gradients, skip connections were introduced to improve the network's information flow and training process. From the previous layer, the feature map's identity mapping is integrated with the output ${H_l}\left( x \right)$ of these skip connections:

(4) $${x_l} = H\left( {{x_{l - 1}}} \right) + {x_{l - 1}}.$$

Moreover, the identity function is integrated using Eq. (4) with the output, ${H_l}$ which can obstruct the network’s data flow.

The network introduces the dense connection to improve the data flow and enhance the skip connection.

(5) $${x_l} = {H_l}\left( {\left[ {{x_{l - 1}},{x_{l - 2}},...,{x_0}} \right]} \right)$$

where (…) signifies the process of concatenation. The dropout, Relu, BN, and convolution layers comprize the nonlinear transformation function. In this network model, the transition layer contains the BN layer, the 2 * 2 average pooling layer, and the 1 * 1 convolutional layer.

## Result and discussions

This section compares the proposed *modus operandi* with the avant-garde techniques and discusses the various consummation measures utilized to assess the method.

The objective of this research was to diagnose various macular pathology diseases accurately. The fundus image dataset was categorized and segmented using the proposed two-phase transfer learning technique. Using the trained Modified ResNet-50 model, several classes of DED disorders are identified. The DenseUNet model using transfer learning is developed to segment different DED disorders. The training is performed by using 70% of the dataset, testing by using 10%, and validation by using 20%. K-fold cross-validation, with k set at five, was used to analyze the experiment results. The model was evaluated on the remaining fifth fold after the proposed structure had been trained using k − 1 = 4 folds. The suggested model’s performance was measured using the performance metric for each k-test (iterations), and the average score was considered.

In this research, transfer learning can be combined with ensemble deep learning-based classification and segmentation models, and this can improve the robustness of the model to data imbalance, as different models may focus on various aspects of the data. The pre-trained model from the source domain has already learned useful features and patterns, which can be valuable for the target domain. Even if the target domain has data imbalance, the transferred knowledge can still provide beneficial effects for identifying patterns related to minority classes. Transfer learning can act as a form of regularization. The model starts closer to a reasonable solution by initialising the model with pre-trained weights. This can help prevent over-fitting on the limited target domain data, which is particularly beneficial when dealing with imbalanced datasets.

The two-phase transfer learning method, developed to classify different DED diseases, uses the fundus images as input. In the proposed network model, the “Stochastic Gradient Descent (SGD)” is used to fine-tune several hyper-parameters to achieve higher performance.

Optimization algorithms are commonly used to perform hyperparameter tuning for deep learning models. Hyperparameter tuning is finding the best combination of hyper-parameters for a classification and segmentation model and dataset to optimize performance. Hyperparameter tuning is an optimization problem. Optimization algorithms are used to search through the defined hyperparameter space in an efficient way to find the combination of hyperparameters that minimizes the objective function. Hyper-parameters, such as learning rates, batch size, number of epochs, and momentum, can be optimized using a gradient-based method like the Stochastic Gradient Descent (SGD) algorithm. These methods involve computing gradients of the objective function concerning the hyper-parameters and iteratively updating them. The SGD algorithm uses a separate validation set for each hyper-parameter set to evaluate the model’s performance. The objective function is computed for each evaluation. The optimization algorithm continues to suggest new sets of hyper-parameters based on the results of previous assessments. A predetermined number of iterations are completed, or the process continues until a convergence criterion is satisfied. After the optimization process, select the set of hyper-parameters for higher performance on the validation or test data. Finally, the classification and segmentation model will be trained using the hyper-parameters chosen on the entire training dataset to obtain the final model. The selected hyperparameter values are a learning rate of 0.1, momentum of 0.8, batch size of 20, and number of epochs of 50.

The specificity, sensitivity, accuracy, confusion matrix, and ROC curve were calculated for each class label for the proposed two-phase transfer learning-based classification and segmentation model.

The suggested device was implemented using an Intel i5 2.60 GHz CPU running Windows 10 and 8 GB of RAM. The investigations were conducted using Python, KERAS, and TensorFlow under the Anaconda3 environment.

### Performance analysis

For the diagnosed DED, various measures were used to determine the false and true categories to analyze the effectiveness of the proposed two-phase transfer learning model. The cross-validation estimator was then employed. The confusion matrix was used to determine the classification performance. The correctly classified samples based on a specific category are represented using true positive (TP) indices. True negative (TN) indices of the confusion matrix, the extra samples that were pertinently related to certain other classes established were contained. Similarly, the false positive (FP) and false negative (FN) indices in the confusion matrix presented how many samples the classifier misestimated. These metrics’ values were determined using Eq. (6) through Eq. (8) formulas.

By dividing the total values of the confusion matrix by the ratio of true positive to actual negative values, Eq. (6) defines accuracy. The factors of the confusion matrix determined the classifier’s performance in the proposed model.

(6) $$Accuracy \;\left({\rm \%} \right) = \displaystyle{{TP + TN} \over {TP + FP + TN + FN}}$$

(7) $$Specificity \; \left( {\rm \%} \right) = \displaystyle{{TN} \over {TN + FP}}$$

(8) $$Sensitivity \; \left( {\rm \%} \right) = \displaystyle{{TP} \over {TP + FN}}$$

(9) $$F1 - Score \;\left({\rm \%} \right) = \displaystyle{{2 \times Precision \times {\mathop{Re}\nolimits} call} \over {Precision + {\mathop{Re}\nolimits} call}}$$

(10) $$Precision \; \left( {\rm \%} \right) = \displaystyle{{TP} \over {TP + FP}}$$

(11) $${\mathop{Re}\nolimits} call = \displaystyle{{TP} \over {TP + FN}}.$$

The Intersection of Union (IOU) and Dice score evaluate the segmentation performance.

(12) $$Dicescore = 2 \times \displaystyle{{Precision \times {\mathop{Re}\nolimits} call} \over {Precision + {\mathop{Re}\nolimits} call}}$$

(13) $$IoU \rightleftarrows score= {{TP} \over{TP+FP+FN}}.$$

The predicted and ground truth labels are used to calculate the evaluation measure.

### Experimental results

**Figure 5:**
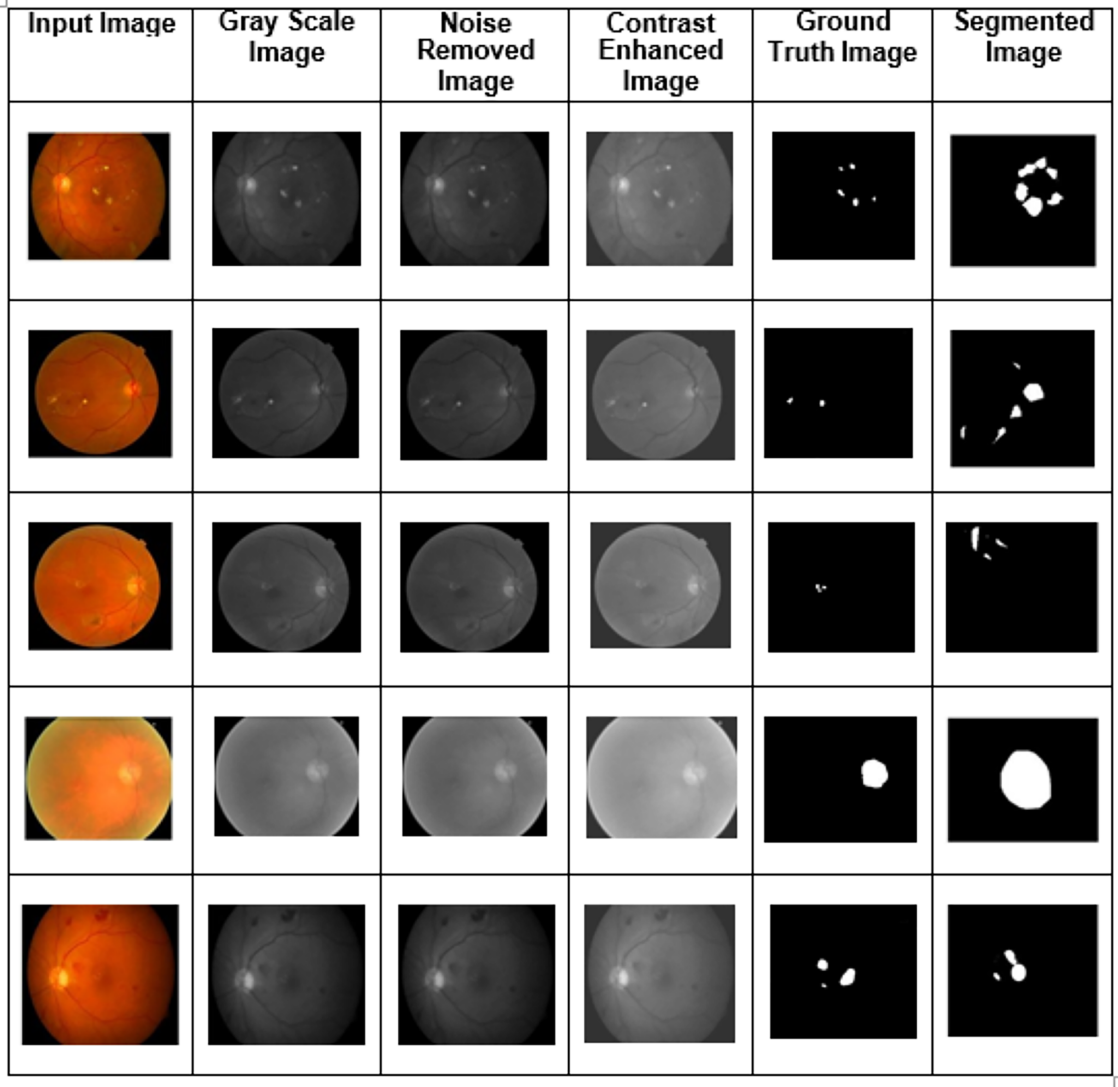
The proposed two-phase transfer learning model’s experimental results.

Training, validation, and testing sets of images are generated from the dataset, and various fundus image types were utilized as training and validation sets. The experiment analysis is performed using 50 epochs. In training and validation, the proposed model achieved the predicted accuracy when all the epochs were complete. Combining features, the output images were constructed using the proposed two-phase transfer learning model. Then, the proposed model classified the fundus images into five significant DED disease classifications: glaucoma, cataract, DME, diabetic retinopathy, and routine. They were utilizing various performance measures. The proposed model's experimental results are shown in Fig. 5.

Table 2 presents the performance metrics derived from the confusion matrices. It demonstrates the proposed model's class-wise specificity, sensitivity, and accuracy. An accuracy of 99.02% was obtained to classify diabetic retinopathy, 98.79% for DME, 99.01% for cataracts and 99.53% for glaucoma classification. It was observed that the performance classification results had significantly improved. The proposed algorithm obtained 99.67% accuracy, 99.73% specificity, 99.54% sensitivity, and 99.02% F1-score. The class-wise performance was assessed using a small sample of the validation set's images. The proposed two-phase transfer learning model effectively performs for multiple classification DED diseases.

**Table 2:** Performance results for classification of the proposed model.

Class label	Accuracy (%)	Sensitivity (%)	Specificity (%)	F1-score (%)
Diabetic retinopathy	99.02	98.32	99.04	98.92
DME	98.79	99.3	99.13	98.28
Cataract	99.01	99.06	99.56	99.64
Glaucoma	99.53	99.01	98.82	99.25

**Figure 6:**
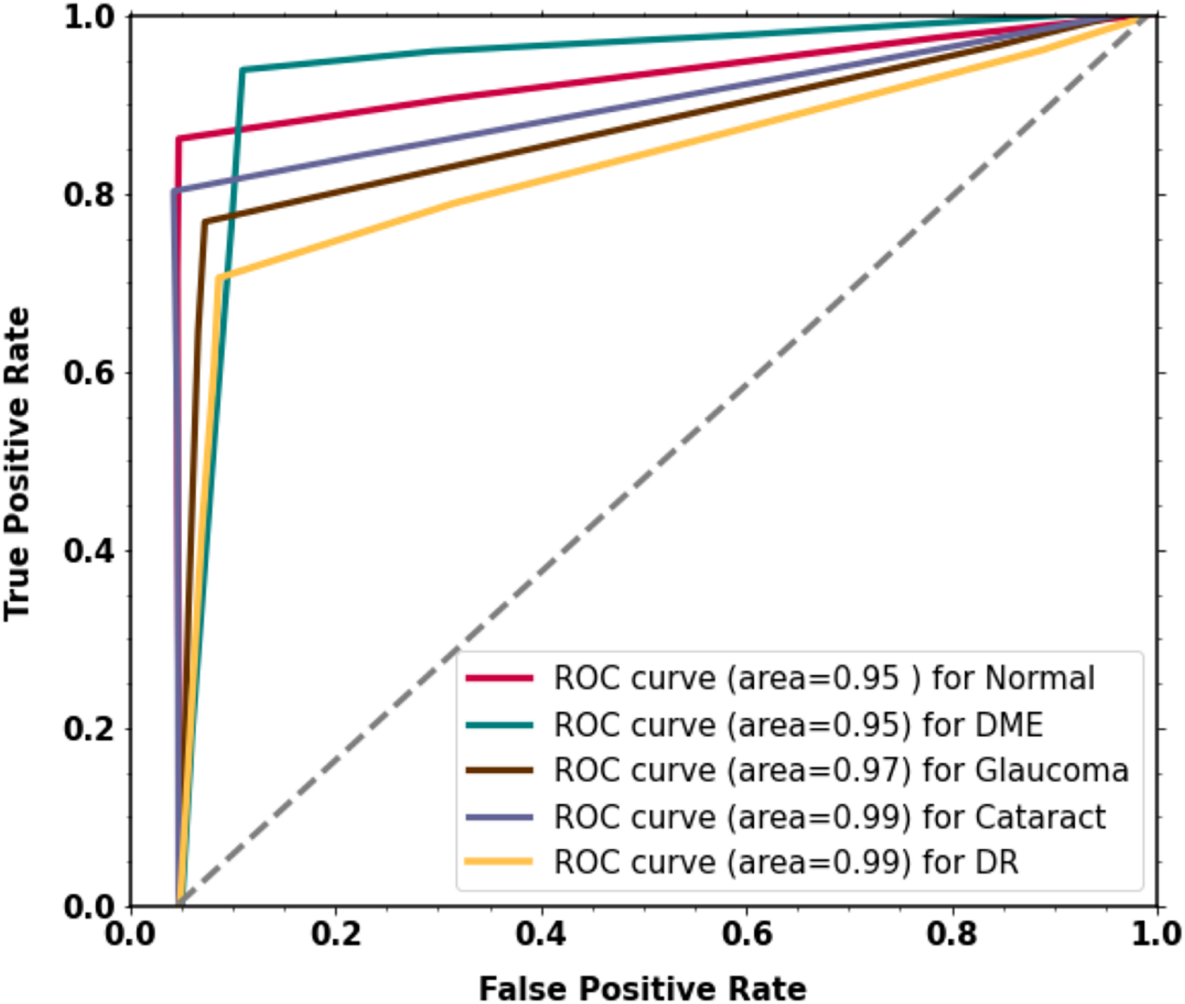
The proposed two-phase transfer learning model’s experimental results.

For the classification of multi-class diabetic diseases, the curve scores for each receiver operating characteristic (ROC) class and area under the curve (AUC) are displayed in Fig. 6. For every class, the model has outstanding AUC scores. The model is deemed acceptable and efficient if it attains the highest AUC of the ROC. The ROC curve was calculated by using the TP and FP metrics. The AUC (ROC) for the proposed methodology is 0.9516.

**Figure 7:**
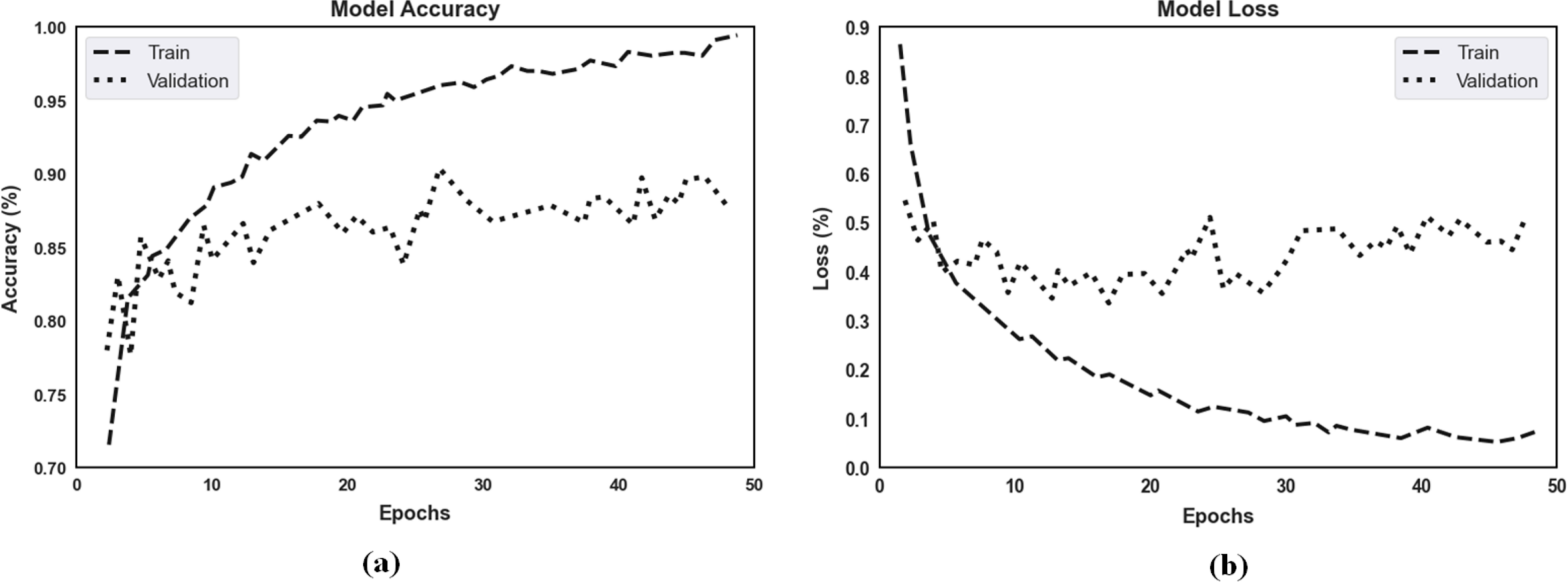
(A) The proposed model’s training accuracy and validation accuracy for classification. (B) The proposed model’s training loss and validation loss for classification.

The proposed model’s accuracy and loss over 50 epochs of training and validation are shown in Fig. 7 for classification. Regarding the experimental results, the highest training accuracy measured was 99.67%, and the proposed model obtained 92.15% accuracy for validation. Losses for the model’s training and validation were 0.011 and 0.069, respectively. Per our experimental results, multiple ocular diseases were accurately identified and classified by proper training in the proposed two-phase transfer learning approach, including diabetic retinopathy, DME, cataract, and glaucoma.

**Figure 8:**
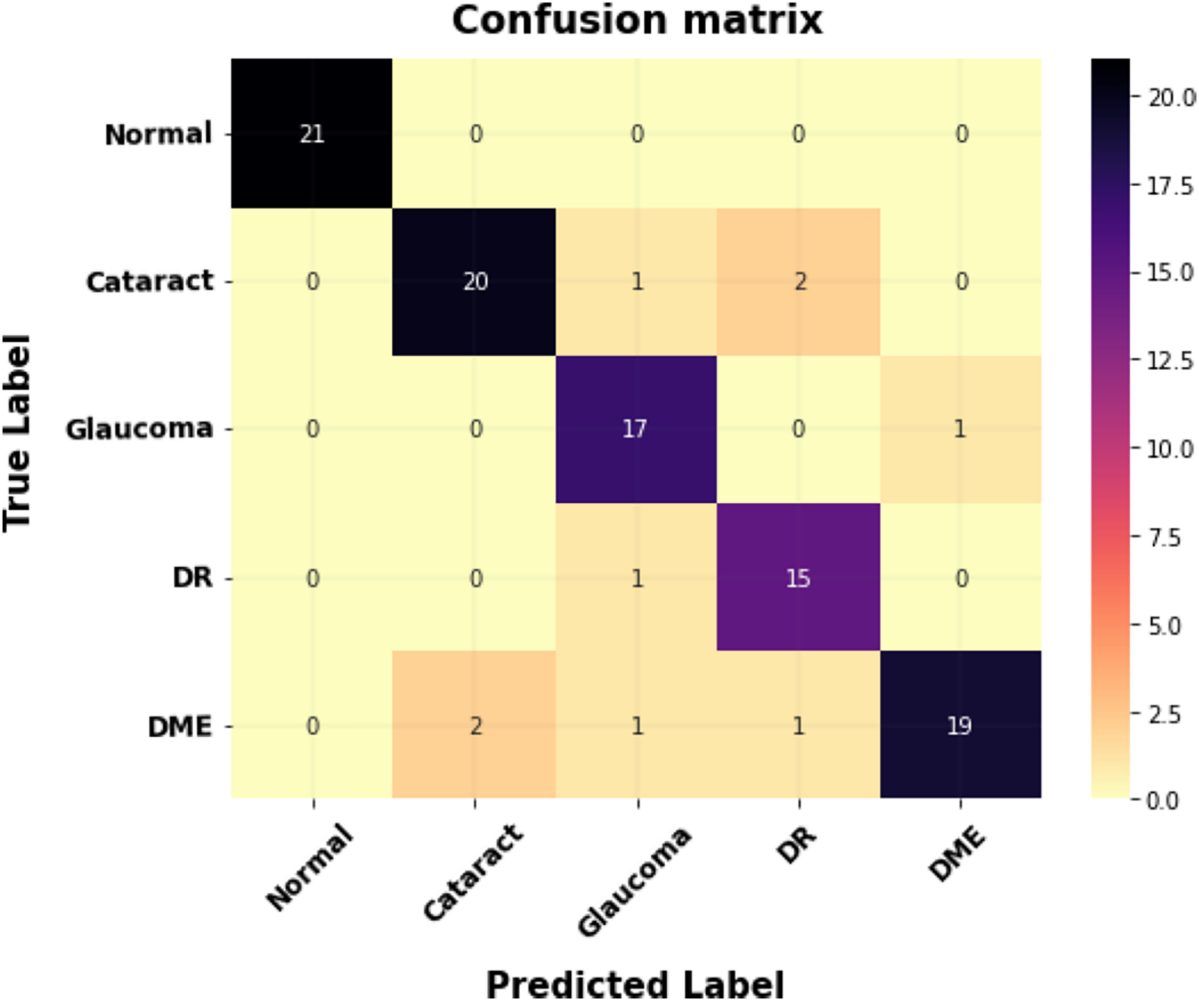
The confusion matrix for multi-class classification.

Figure 8 shows the proposed model’s confusion matrix for classification. The X-axis indicates the proposed system outputs, and the Y-axis indicates the ground truth values.

Two evaluation metrics, IoU score and Dice score, are used to evaluate the segmentation results of each testing data for multiple classes of fundus images for the segmented maps per pixel and compared with the original ground truth. The segmentation results for multiple DED diseases using fundus images are given in Table 3. For image segmentation, it is noted that the proposed transfer learning-based DenseUNet model produced the overall highest Dice score of 95.50% and IoU score of 95.01%on the test set fundus images for different diabetic eye diseases.

**Table 3:** Performance results for segmentation.

Classes	Dice score (%)	IOU score (%)
Normal	96.34	94.21
Cataract	95.5	95.35
Glaucoma	92.05	90.8
DR	95.5	95.5
DME	95.26	95.01

Compared to datasets used to train models from scratch, datasets related to diabetic eye disease may be smaller. The proposed two-phase transfer learning reduces the chance of overfitting by improving the smaller dataset and utilizing the pre-trained model’s generalization abilities. This method frequently produces more reliable models and enables better use of the available data. Fine-tuning the network layers usually results in faster convergence during training on the diabetic eye disease dataset, as the initial layers of the pre-trained model have already learned general features from a dataset. This may shorten the amount of time needed to train the model. Images of diabetic eye diseases might differ significantly in quality, light, and patient features. Transfer learning facilitates the model’s acquisition of robust features that overcome this kind of unpredictability, improving classification performance. Images of diabetic eye diseases may differ in pathology, texture, and colour. Transfer learning allows the segmentation model to adapt to these variances and produce more accurate segmentation results across various image features by fine-tuning on a smaller dataset.

### Time complexity analysis

The estimated processing time (for this study) is shown in Table 4, and it is an essential factor in the image retrieval process. Each image’s processing, training, and testing times comprise the complete time procedure. Pre-processing, classification, and segmentation processes comprise the processing time; reading the image is the first step, and segmentation is the last. Likewise, the training time for each network is the amount of time required to train the complete dataset. Each network’s prediction and vote constitute the only testing time. However, this research estimated a processing time of around 10 s, which is comparatively quicker than state-of-the-art methods. The average power consumption to compute each sample was recorded as 25.656 W.

**Table 4:** The entire image processing time of the research.

Item	Time complexity (ms)	Other info
Processing time (pre-processing + classification + segmentation)	9.56356	Per image
Training time	0.437288	Per dataset
Testing time	0.037923	Per image

### Comparison results

Using the two evaluation metrics, including the Dice score and IoU score, the segmentation results of each testing data were compared to the original ground truth for the segmented maps per pixel, as shown in Table 5. For multi-class diabetic eye illnesses, it should be emphasized that the proposed transfer learning-based DenseU-Net model generated the highest overall dice score of 96.34%. Additionally, across many classes of diabetic eye disorders, the suggested model had the most significant overall IoU Score of 91.21%. Regarding the Dice score and IoU score, the proposed transfer learning-based DenseU-Net model performs better than the other related segmentation models (Wang et al., 2019; Wang, Li & Cheng, 2023) based on the comparative results.

**Table 5:** Results of comparison on the testing datasets between the proposed transfer learning-based DenseUNet model and the related segmentation models.

Model	Dice score (%)	IoU score (%)
FFU-Net	77.55	63.33
U-Net	93.9	89.01
EE-Unet	92.2	93.1
Modified U-Net	85.01	75.28
Proposed transfer learning based DenseU-Net model	95.5	95.01

The proposed method’s classification results were compared with those from prior research using fundus images. The comparison results in Table 6 were used to conduct a detailed analysis of the proposed two-phase transfer learning model in terms of specificity, sensitivity, and accuracy.

**Table 6:** Analysis of the proposed methodology in comparison to cutting-edge methods.

Reference	Classifications	Technique	Accuracy (%)	Sensitivity (%)	Specificity (%)
Gour & Khanna (2021)	DR, glaucoma, AMD, myopia, hypertension, and cataract	Transfer learning based VGG16-SGD	96.49	94.02	95.22
Demir & Taşcı (2021)	DR, glaucoma, AMD, myopia, hypertension, and cataract	R-CNN+LSTM	89.54	85.00	96.85
Nazir et al. (2021)	DR and DME	CenterNet	98.01	96.72	96.30
Sarki et al. (2021b)	Normal, DR, DME, glaucoma, and cataract	CNN	81.33	95.32	94.32
Kim et al. (2021)	DR, RVO, RP, MH, RRD, ERM, dAMD, and nAMD	Inception v3	98.08	96.04	97.53
Muthukannan (2022)	Normal, DR, DME, glaucoma, and cataract	FPOA-CNN	98.30	98.28	–
**Proposed research**	**Normal, DR, DME, glaucoma, and cataract**	**The two-phase transfer learning approach**	**99.67**	**99.54**	**99.73**

Gour & Khanna (2021) developed a Transfer learning-based VGG16-SGD model, and 96.49% accuracy was achieved by this model for multiple retinal disease classification. Demir & Taşcı (2021) provided a hybrid deep learning-based R-CNN+LSTM model for the multi-classification of retinal diseases, which classified the fundus image to DR, glaucoma, AMD, myopia, hypertension, and cataract. Nazir et al. (2021) utilized the CenterNet model and achieved 98.01% accuracy for multi-class DED disease classification. An accuracy of 81.33% was attained by Sarki et al. (2021b) for the multiclassification of normal, DR, DME, glaucoma, and cataracts using CNN. The inception v3 model was proposed by Kim et al. (2021) for multiple macular disease classification. It classified the retinal image into diabetic retinopathy (DR), retinal vein occlusion (RVO), retinitis pigmentosa (RP), macular hole (MH), rhegmatogenous retinal detachment (RRD), epiretinal membrane (ERM), and neovascular or dry age-related macular degeneration (nAMD or dAMD), it achieved 98.08% accuracy. Muthukannan (2022) proposed an FPOA-CNN approach for multi-classification of ocular diseases, which achieved an accuracy of 98.30%. The proposed two-phase transfer learning-based modified ResNet-50 and DenseUNet model achieved better results than the recent existing models. The multiple retinal diseases are correctly classified, and the detected tumour region is accurately segmented using the proposed approach. The key advantages of the proposed classification network model are that it prevents overfitting and has no detrimental effects on network performance because of the classification process.

**Figure 9:**
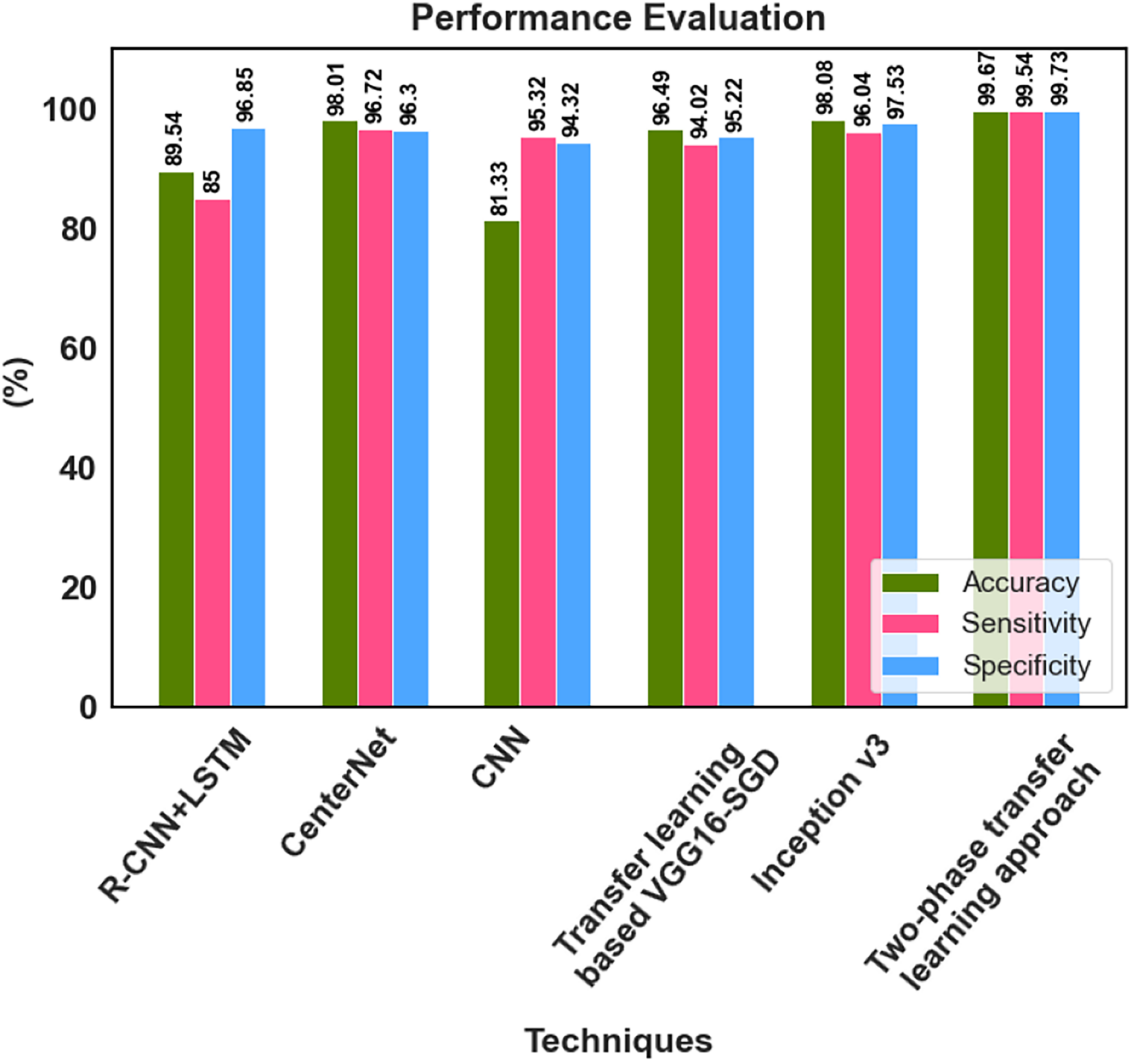
Performance analysis graph.

The suggested model accurately extracts the essential features corresponding to the diversity of diseases across different scales, enhancing classification performance. He developed a deep learning-based hybrid model to classify DED disease that outperformed the state-of-the-art models. The performance of 99.73% specificity, 99.54% sensitivity and 99.67% accuracy is achieved by the proposed approach. The classification accuracy enabled automatic diagnosis and pre-screening of various DED diseases. The anomaly patterns were recognized effectively by the proposed network model, and discriminative sequences were produced, which assisted in identifying DED diseases. According to the experimental results, the fundus images for classifying various retinal diseases, the proposed two-phase transfer learning approach, are priceless tools for healthcare providers. Figure 9 shows the performance comparison graph for proposed and existing methods.

This research is subject to certain limitations. Two different resolutions for the fundus images in our study were used. The images are pre-processed to the exact resolution of 512 pixels × 512 pixels to reduce the difference and then used for AI learning. Even though the resolutions of the two types of images were identically matched by pre-processing, it is possible that this step did not entirely remove the differences between them. Secondly, while the reading time was reduced by half with the aid of AI technology, the performance did not improve as much. However, it has been demonstrated that this research can aid in the differential diagnosis of many retinal disorders using AI deep learning.

## Conclusion

This research reveals an elaborative and scientific use of two-phase transfer learning approaches for accurately categorizing and recognizing ocular disease regions, including typical diabetic retinopathy, DME, cataracts, and glaucoma. The proposed approach was tested using a large dataset of pre-processed fundus images with enhanced contrast and noise removal. The Modified ResNet-50 was used for five ocular diseases’ classifications, including typical, diabetic retinopathy, DME, cataract, and glaucoma, with high specificity, sensitivity, and accuracy, reducing detection time and epochs. Then the defective regions of ocular diseases are segmented using the transfer learning-based DenseUNet model. The proposed two-phase transfer learning-based Modified ResNet-50-DenseUNet model effectively classified and segmented the fundus images, outperforming existing models considerably per the experimental results with 99.67% accuracy. While avoiding human subjectivity, the developed system serves as an auxiliary diagnosis reference and allows for standardizing labour-intensive eye-screening procedures. We also want to expand our design or create a new convolutional neural network to achieve more precise outcomes in automated and simultaneous segmentation tasks for various retinal disorders, including hard and soft exudates, haemorrhages, and micro-aneurysms.

## Additional Information and Declarations

### Data Availability

The following information was supplied regarding data availability: The code is available in figshare: Krishna, M. Vamsi; B. Srinivasa Rao, Dr. (2024). Code for the research journal "Detection and Diagnosis of Diabetic Eye Diseases using Two Phase Transfer Learning Approach". figshare. Dataset. https://doi.org/10.6084/m9.figshare.22760705.v2. The Messidor dataset is available at Kaggle: https://www.kaggle.com/datasets/hanhan2010/messidor. The Messidor 2 is dataset is available at Kaggle: https://www.kaggle.com/datasets/google-brain/messidor2-dr-grades. The DRISHTI-GS dataset is available at Kaggle: https://www.kaggle.com/datasets/lokeshsaipureddi/drishtigs-retina-dataset-for-onh-segmentation. The Cataract dataset is available at Kaggle: https://www.kaggle.com/datasets/gunavenkatdoddi/eye-diseases-classification.
